# Development and Evaluation of Stable Sugarcane Mosaic Virus Mild Mutants for Cross-Protection Against Infection by Severe Strain

**DOI:** 10.3389/fpls.2021.788963

**Published:** 2021-12-17

**Authors:** Xiao-Jie Xu, Qing Zhu, Shao-Yan Jiang, Zhi-Yong Yan, Chao Geng, Yan-Ping Tian, Xiang-Dong Li

**Affiliations:** Shandong Province Key Laboratory for Agricultural Microbiology, Laboratory of Plant Virology, Department of Plant Pathology, College of Plant Protection, Shandong Agricultural University, Tai’an, China

**Keywords:** cross-protection, helper component-proteinase, RNA silencing suppression, spontaneous mutation, virulence, sugarcane mosaic virus

## Abstract

Sugarcane mosaic virus (SCMV; genus *Potyvirus*) induces maize dwarf mosaic disease that has caused serious yield losses of maize in China. Cross-protection is one of the efficient strategies to fight against severe virus strains. Although many mild strains have been identified, the spontaneous mutation is one of the challenging problems affecting their application in cross-protection. In this study, we found that the substitution of cysteine (C) at positions 57 or 60 in the zinc finger-like motif of HC-Pro with alanine (A; C57A or C60A) significantly reduced its RNA silencing suppression activity and SCMV virulence. To reduce the risk of mild strains mutating to virulent ones by reverse or complementary mutations, we obtained attenuated SCMV mutants with double-mutations in the zinc finger-like and FRNK motifs of HC-Pro and evaluated their potential application in cross-protection. The results showed that the maize plants infected with FKNK/C60A double-mutant showed symptomless until 95 days post-inoculation and FKNK/C60A cross-protected plants displayed high resistance to severe SCMV strain. This study provides theoretical and material bases for the control of SCMV through cross-protection.

## Introduction

Maize dwarf mosaic (MDM) is one of the most serious viral diseases, which threatens the safety of maize production in all maize-growing regions of China (Gao et al., 2011; Wu et al., 2012; Xie et al., 2016). Sugarcane mosaic virus (SCMV) belonging to the genus *Potyvirus* is the prevalent virus inducing MDM in China (Jiang and Zhou, 2002; Yan et al., 2016; Chen H. et al., 2017). However, little is known about effective measures to prevent SCMV.

Cross-protection is an environmentally safe method to control plant viral diseases (Pechinger et al., 2019) and has been used to control multiple plant viruses in the laboratory or field (Rast, 1972; Krstic et al., 1995; Cong et al., 2019; Cheng et al., 2020). Mild viruses can protect plants from subsequent infection of the same or closely related severe strains (Zhang and Qu, 2016; Zhang et al., 2018). However, screening mild mutants for cross-protection is a time-consuming task by traditional treating with nitrous acid, ultraviolet irradiation, or heat (Yeh and Cheng, 1989; Yang et al., 2002). Reverse genetics provides a faster and more effective way for screening mild mutants (Kung et al., 2014; Huang et al., 2019; Tuo et al., 2020). The stability of the mild mutants is one of the most important factor affecting the application of cross-protection (Ziebell and MacDiarmid, 2017). The reverse or complementary mutations increase the risk of mild strains becoming virulent ones (Sherwood, 1987; Loebenstein and Carr, 2006; Xu et al., 2020). Compared with that of viral mutants with a single mutation, the possibility of reversion to virulent ones for mutants carrying multiple mutations was lower (Maassab and DeBorde, 1985; Tosh et al., 2008; Liu et al., 2017). Therefore, mild strains with double or more mutations are preferred in cross-protection (Lin et al., 2007; Cong et al., 2019; Tuo et al., 2020). In addition, cross-protection only works well between closely related viruses (Matthews, 1949; Pechinger et al., 2019), and the local dominant strains should be used to screen mild mutants for cross-protection.

Potyviruses encode two polyproteins which are cleaved into 11 mature proteins by three virus-encoded proteinases (Chung et al., 2008; Olspert et al., 2015). Multifunctional helper component-proteinase (HC-Pro) of potyvirus is involved in RNA silencing suppression, aphid transmission, viral movement and virulence (Pirone and Blanc, 1996; Sáenz et al., 2002; Valli et al., 2014; Ivanov et al., 2016). Previous studies have revealed the role of HC-Pro FRNK motif in potyvirus virulence (Shiboleth et al., 2007; Gao et al., 2012; Kung et al., 2014; Xu et al., 2020). The N-terminal domain of potyviral HC-Pro contains a highly conserved cysteine (C)-rich region that belongs to the zinc finger-like motif (Robaglia et al., 1989). Mutations in the conserved C residues within the zinc finger-like motif have a strong debilitating effect on the self-interaction activity of PVY HC-Pro (Urcuqui-Inchima et al., 1999). The mutation of C at position 310 in HC-Pro zinc finger-like motif to serine (S) has profound effects on the virulence of tobacco vein mottling virus (TVMV) (Atreya and Pirone, 1993). So far, there has been no report on the role of the conserved C residues in the zinc finger-like motif of SCMV HC-Pro in its RNA silencing suppression (RSS) activity and the virulence of SCMV that mainly infects monocot crops.

In this study, we found that the zinc finger-like motif of HC-Pro was involved in its RSS activity and SCMV virulence. Our previous study has shown that the attenuated SCMV mutant with single-mutation in HC-Pro FRNK motif could protect maize plants from severe strain, while a spontaneous mutation restored its virulence (Xu et al., 2020). To reduce this risk, we obtained attenuated SCMV mutants with double-mutations in HC-Pro zinc finger-like and FRNK motifs, and evaluated their potential in cross-protection. This study provided theoretical and practical bases for the control of SCMV via cross-protection.

## Materials and Methods

### Plant Growth and Virus Inoculation

Plants of maize (*Zea mays*) inbred line B73, *Nicotiana benthamiana*, and GFP-expressing *N. benthamiana* (16C) were cultivated in a growth chamber with 16 h light (24°C) and 8 h dark (22°C) cycles.

The pSCMV-based constructs were inoculated onto leaves of the three-leaf staged maize plants as described previously (Xu et al., 2020). Crude extracts from the maize leaves infected with SCMV carrying *gfp* reporter gene (SCMV-GFP) or SCMV mutants were ground in 20 mM phosphate-buffered saline (pH 7.2) and inoculated onto maize leaves via mechanical rub. These experiments were repeated thrice independently.

### Plasmid Construction

The infectious clone based on SCMV-BJ isolate (pSCMV) was kindly provided by Professor Yule Liu from Tsinghua University, China. Site-directed mutagenesis was performed as described previously (Liu and Naismith, 2008). The primers used for mutation were listed in Supplementary Table 1. For transient expression in 16C *N. benthamiana* leaves, the full-length coding sequence of SCMV HC-Pro or its mutants were ligated into pBin121 vector between *Bam*HI and *Sac*I restriction sites.

### RNA Silencing Suppression Assay

Plasmids pBin-GUS and the plasmids expressing wild-type SCMV HC-Pro (pBin-HC) or its mutants were transformed into *Agrobacterium* cells, respectively. The transformed *Agrobacterium* cultures were grown overnight in the Luria-Bertani culture medium containing 50 μg/mL kanamycin and 50 μg/mL rifampicin followed by 3 h of incubation in an induction buffer [10 mM MgCl_2_, 150 μM acetosyringone and 10 mM 2-(N-Morpholino) ethane sulfonic acid (MES)] at room temperature. *Agrobacterium* cultures (OD_600_ = 0.3) were individually mixed with *Agrobacterium* cells harboring plasmid pBin-GFP in a ratio of 1:1 before infiltration into 16C *N. benthamiana* leaves. Green fluorescence was photographed using a Canon 800D camera under UV light. The experiments were repeated thrice independently.

### RNA Extraction and Quantitative Real-Time Reverse Transcription Polymerase Chain Reaction

Total RNA was extracted from maize and *N. benthamiana* 16C leaf tissues, and the first-strand cDNA for reverse transcription polymerase chain reaction (RT-PCR) was synthesized as described previously (Xu et al., 2020). The Quantitative Real-Time RT-PCR (qRT-PCR) was performed using ChamQ SYBR qPCR Master Mix (Vazyme, Nanjing, China) on a PCR machine (LC96, Roche, Basel, Switzerland). The house-keeping genes including maize *ZmUbi* gene (GenBank accession: **XM_008647047**) and *N. benthamiana actin* gene (GenBank accession: **AY179605**) were used as internal controls for qRT-PCR (Gao et al., 2012; Chen H. et al., 2017) (Supplementary Table 1). Each qRT-PCR was performed with three biological replicates and three technical replicates.

### Enzyme-Linked Immunosorbent Assay

The maize upper non-inoculated leaves were extracted with coating buffer (15 mM Na_2_CO_3_ and 35 mM NaHCO_3_, pH 9.6), added to the microplate wells, and incubated at 37°C for 4 h. The rabbit polyclonal antibody against SCMV CP was used as the primary antibody. Alkaline phosphatase-conjugated goat anti-rabbit IgG (1:50000, v/v) was used as the secondary antibody. Furthermore, the absorbance value at 405 nm was measured using a Multi-function Microplate Reader (BioTek Synergy™ Mx, Winooski, VT, United States). The ELISA was performed with three biological replicates and repeated thrice independently.

### Western Blotting

Western blotting was performed as described previously (Sun and Suzuki, 2008). The primary antibodies against SCMV CP, SCMV HC-Pro and GFP were prepared in the Laboratory of Plant Virology, Shandong Agricultural University (Wang et al., 2014; Xu et al., 2018, 2019). The horseradish peroxidase-conjugated goat anti-rabbit immunoglobulin G (IgG) was used as the secondary antibody (Sigma-Aldrich, St. Louis, MO, United States). Quantification of SCMV CP and GFP accumulation levels were estimated using ImageJ software (Wyrsch et al., 2015). The samples from three biological replicates were detected separately.

### Cross-Protection Assay

Cross-protection experiments was conducted as described in our previous study with minor improvement (Xu et al., 2020). The time between the inoculation of mild strain and wild-type SCMV-GFP were set to 15 and 20 days, respectively. Western blotting was used to determine the accumulation levels of wild-type SCMV-GFP at 20 days after the challenge inoculation. Three independent experiments were carried out. *Agrobacterium* cells carrying empty vector pCB301-Rz were used as control.

### Genetic Stability Assay

The genetic stability of SCMV mutants was tested through successive passages in maize plants. The crude extracts from upper non-inoculated maize leaves infected with SCMV mutants were used for inoculating healthy maize leaves. The mutants were successively transferred for four generations in maize plants at 15 day intervals. The HC-Pro coding sequences of the fourth generation SCMV progeny in maize plants were sequenced. In addition, the HC-Pro coding sequences from the SCMV progeny in maize plants infected with SCMV mutants were sequenced at 30 days post inoculation (dpi) and 60 dpi, respectively.

## Results

### Mutations on the Conserved C^57^ and C^60^ of Helper Component-Proteinase Reduced Its RNA Silencing Suppression Activity and Sugarcane Mosaic Virus Virulence

**FIGURE 1:**
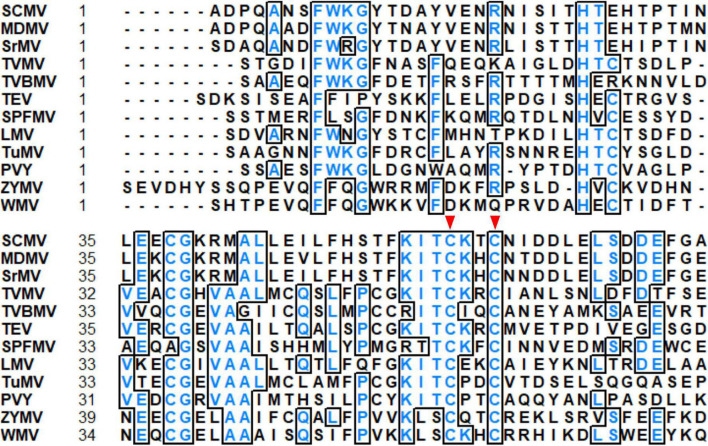
Alignment of partial HC-Pros sequences of 12 potyviruses. The highly conserved cysteine residues at positions 57 and 60 (C^57^ and C^60^) in SCMV HC-Pro were pointed by the red triangles. The analysis was performed with the sequences of SCMV (GenBank accession: AY042184), maize dwarf mosaic virus (MDMV, GenBank accession: AJ001691), sorghum mosaic virus (SrMV, GenBank accession: U57358), tobacco vein mottling virus (TVMV, GenBank accession: NP_734329), tobacco vein banding mosaic virus (TVBMV, GenBank accession: EF219408), tobacco etch virus (TEV, GenBank accession: DQ986288), sweet potato feathery mottle virus (SPFMV, GenBank accession: KU511268), lettuce mosaic virus (LMV, GenBank accession: AJ306288), turnip mosaic virus (TuMV, GenBank accession: AB093596), potato virus Y (PVY, GenBank accession: AB185833), zucchini yellow mosaic virus (ZYMV, GenBank accession: AY188994) and watermelon mosaic virus (WMV, GenBank accession: AB218280).

**FIGURE 2:**
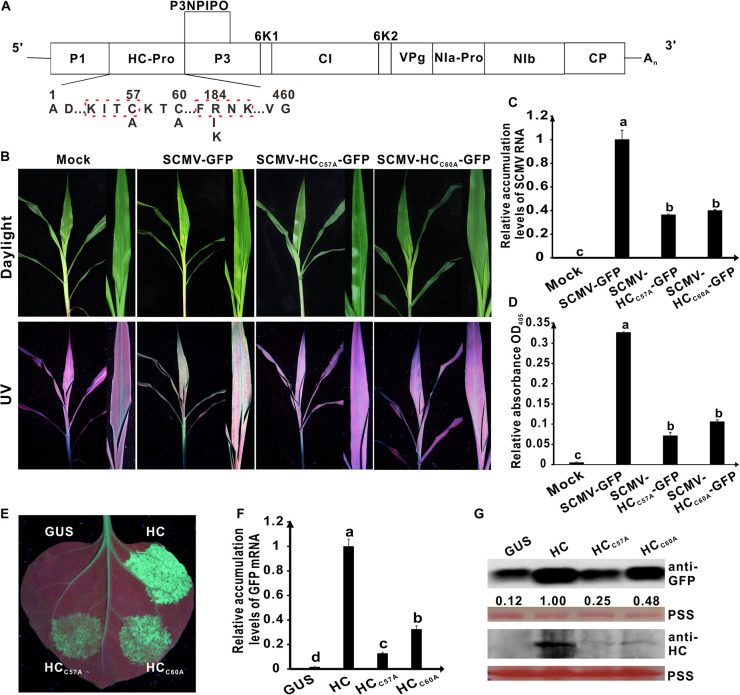
Effects of the mutations in the conserved C^57^ and C^60^ of wild type HC-Pro on its RNA silencing suppression activity and virulence of SCMV. **(A)** Genetic map of SCMV, showing the all mutations in HC-Pro. The numbers above the sequence indicate their position in SCMV HC-Pro and the letters below the sequence showed the substituted residues at that position. The highly conserved KITC and FRNK motifs in SCMV HC-Pro were marked by the red dotted boxs. **(B)** Symptoms of SCMV and two SCMV mutants in maize plants at 10 days post inoculation (dpi). The conserved C^57^ and C^60^ in wild type SCMV HC-Pro were mutated to A residues in HC-Pros of SCMV-HC_*C*57*A*_-GFP and SCMV-HC_*C*60*A*_-GFP, respectively. Mock, the maize plants inoculated with the empty vector pCB301-Rz. SCMV-GFP, the maize plants infected with wild type SCMV with *gfp* reporter gene. **(C)** The wild type and mutant SCMV RNA accumulation levels in the upper leaves of maize plants at 10 dpi. **(D)** ELISA analysis of the wild type and mutant SCMV accumulation levels in the upper leaves of maize plants at 10 dpi. **(E)** The wild type and mutants HC-Pro RSS activity in *Agrobacterium* co-infiltration assay. The *N. benthamiana* 16C leaves were infiltrated with a mixture of *Agrobacterium* cultures carrying pBin-GFP and either wild type or mutant HC-Pro and photographed under long-wavelength UV light at 3 days post agroinfiltration (dpai). The conserved C residues in wild type HC-Pro (HC) were mutated to A residues in HC_*C*57*A*_ and HC_*C*60*A*_, respectively. The GUS was used as a negative control. **(F)** The GFP mRNA accumulation levels in agroinfiltrated 16C leaf patches. **(G)** Western blotting analysis of the accumulation levels of GFP and HC in agroinfiltrated leaf patches of 16C. The ponceau S staining (PSS) shows sample loadings. Band intensities were measured using the ImageJ software. Numbers indicate the accumulation levels of SCMV CP normalized to PSS staining. Error bars indicate the means ± standard deviation of three replicates. Statistical significance was determined by employing *Tukey* multiple range test for between-group comparisons. Different letters indicate significant differences (*P* < 0.05). The same below. The experiments were repeated thrice independently.

The N-terminus of HC-Pro contains a highly conserved cysteine (C)-rich region, which belongs to the zinc finger-like motif. By alignment of HC-Pro amino acid sequences of 12 potyviruses, we found that C at positions 57 and 60 (C^57^ and C^60^) in the zinc finger-like motif of SCMV HC-Pro were highly conserved (Figure 1). Furthermore, C^57^ was located in the KITC motif involved in aphid transmission (Figures 1, 2A). Then we investigated the role of C^57^ and C^60^ in HC-Pro RSS activity and SCMV virulence.

To explore the role of amino acid residues C^57^ and C^60^ in determining SCMV virulence, we carried out the site-directed mutagenesis using primers listed in Supplementary Table 1. The resulting plasmids pSCMV-HC_*C*57*A*_-GFP and pSCMV-HC_*C*60*A*_-GFP were agroinfiltrated to *N. benthamiana* leaves, respectively, and mechanically inoculated to maize plants 3 days later. Five plants were inoculated for each treatment. The corresponding amino acid of C^57^ or C^60^ was alanine (A) in HC-Pro derived from the progeny of SCMV mutants (Figure 2A). At 10 dpi, wild-type SCMV with the *gfp* reporter gene (SCMV-GFP) caused severe mosaic symptoms and induced strong GFP fluorescence under UV light in the upper non-inoculated leaves of maize. Compared with the wild-type SCMV-GFP, the symptoms caused by SCMV-HC_*C*57*A*_-GFP and SCMV-HC_*C*60*A*_-GFP were attenuated significantly under daylight and the fluorescence intensity of GFP markedly decreased under UV light in the maize upper leaves at 10 dpi (Figure 2B). Results of qRT-PCR showed that compared with the wild-type SCMV-GFP, SCMV RNA accumulation levels decreased by about 60% for SCMV-HC_*C*57*A*_-GFP and SCMV-HC_*C*60*A*_-GFP (Figure 2C). Enzyme-linked immunosorbent assay results showed that the accumulation levels of SCMV-HC_*C*57*A*_-GFP and SCMV-HC_*C*60*A*_-GFP were significantly (*P* < 0.05) lower than that of wild-type SCMV-GFP (Figure 2D). These results indicated that C^57^ and C^60^ of HC-Pro played an important role in determining SCMV virulence in maize plants.

To investigate the role of amino acid residues C^57^ and C^60^ in SCMV HC-Pro RSS activity, we cloned the HC-Pro coding sequence into pBin121 vector. The resulting plamids was named pBin-HC. The codons encoding C^57^ and C^60^ in pBin-HC were mutated to codon encoding Alanine (A) using primers listed in Supplementary Table 1, and the resulting plasmids were named pBin-HC_*C*57*A*_ and pBin-HC_*C*60*A*_. They were transformed into *Agrobacterium* cells and then individually mixed with *Agrobacterium* harboring plasmid pBin-GFP in a ratio of 1:1. The mixtures were infiltrated into the fully expanded leaves of *N. benthamiana* 16C plants. The *Agrobactium* harboring plasmid pBin-GUS was used as a negative control. At 3 days post agroinfiltration (dpai), no GFP fluorescence was observed in the patches expressing GUS; GFP fluorescence was apparent in the patch expressing HC; however, GFP fluorescence in the patches expressing HC_*C*57*A*_ or HC_*C*60*A*_ was significantly reduced compared with wild type HC-Pro (Figure 2E). qRT-PCR showed that GFP mRNA accumulated only up to about 20%–30% of wild-type HC-Pro for HC_*C*57*A*_ and HC_*C*60*A*_ (Figure 2F). Western blotting results showed that GFP accumulation levels in 16C leaf patches expressing HC-Pro mutants HC_*C*57*A*_ and HC_*C*60*A*_ were significantly lower than that of wild-type HC-Pro (Figure 2G). Interestingly, the HC-Pro accumulation levels of mutants HC_*C*57*A*_ and HC_*C*60*A*_ were significantly lower than that of wild-type HC-Pro in 16C leaf patches (Figure 2F).

These results showed that the C^57^ and C^60^ residues in HC-Pro zinc finger-like motif played a critical role in SCMV virulence and HC-Pro RSS activity in plants.

### Three Sugarcane Mosaic Virus Mutants With Double-Mutations in Helper Component-Proteinase Displayed Reduced Virulence

**FIGURE 3:**
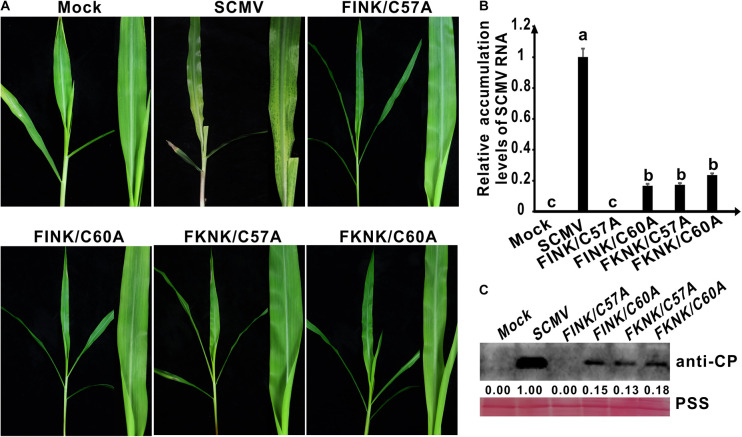
Symptoms and accumulation levels of SCMV double-mutants in maize plants. **(A)** Symptoms of wild-type SCMV and its double-mutants in maize plants at 10 dpi. Mock, the maize plants inoculated with the empty vector pCB301-Rz. SCMV, the maize plants infected with wild-type SCMV. FINK/C60A, the conserved C^60^ was mutated to A residues and R^184^ was mutated to I residues in SCMV HC-Pro. FKNK/C57A, the conserved C^57^ were mutated to A residues and R^184^ were mutated to K residues in SCMV HC-Pro. FKNK/C60A, the conserved C^60^ was mutated to A residues and R^184^ were mutated to K residues in SCMV HC-Pro. **(B)** The wild-type and mutant SCMV RNA accumulation levels in the upper leaves of maize plants at 10 dpi. **(C)** Western blotting analysis of the wild type and mutant SCMV accumulation levels in the upper leaves of maize plants at 10 dpi. The ponceau S staining (PSS) shows sample loadings. Band intensities were measured using the ImageJ software. Numbers indicated the accumulation levels of SCMV CP normalized to PSS staining. The experiments were repeated thrice independently. The statistical analyses as above.

In our previous study, we found that arginine (R) at position 184 (R^184^) of the FRNK motif was also involved in SCMV virulence in maize plants (Xu et al., 2020). Double-mutant plasmids pSCMV-HC_*FINK/C*57*A*_, pSCMV-HC_*FINK/C*60*A*_, pSCMV-HC_*FKNK/C*57*A*_ and pSCMV-HC_*FKNK/C*60*A*_ were obtained according to the above described method using the primers listed in Supplementary Table 1. The progeny of four SCMV double-mutants as follows: C^57^ or C^60^ to A (C57A or C60A) and R^184^ to I or K (FINK or FKNK) of SCMV HC-Pro, respectively (Figure 2A). At 10 dpi, the upper leaves of maize plants inoculated with wild-type SCMV showed severe mosaic and yellowing symptoms; whereas the maize plants inoculated with four SCMV double-mutants presented as symptomless (Figure 3A). Results of qRT-PCR showed that SCMV RNA accumulated up to 20% of wild type SCMV for FINK/C60A, FKNK/C57A and FKNK/C60A mutants, while the accumulation levels of SCMV RNA in maize plants inoculated with FINK/C57A and pCB301 were similar and significantly lower (*P* < 0.5) than that of the other three SCMV double-mutants (Figure 3B). Western blotting results showed that FINK/C60A, FKNK/C57A and FKNK/C60A mutants accumulated to similar level, but significantly lower than that of wild type SCMV (Figure 3C). And in the upper leaves of maize plants inoculated with FINK/C57A and pCB301, SCMV CP could not be detected (Figure 3C). Thus, the double-mutant FINK/C60A, FKNK/C57A and FKNK/C60A, but not FINK/C57A, were capable of systemically infecting maize plants and were candidates for eliciting cross-protection.

### Attenuated Double-Mutant FKNK/C60A Could Protect Maize Plants Against Severe Strain

**FIGURE 4:**
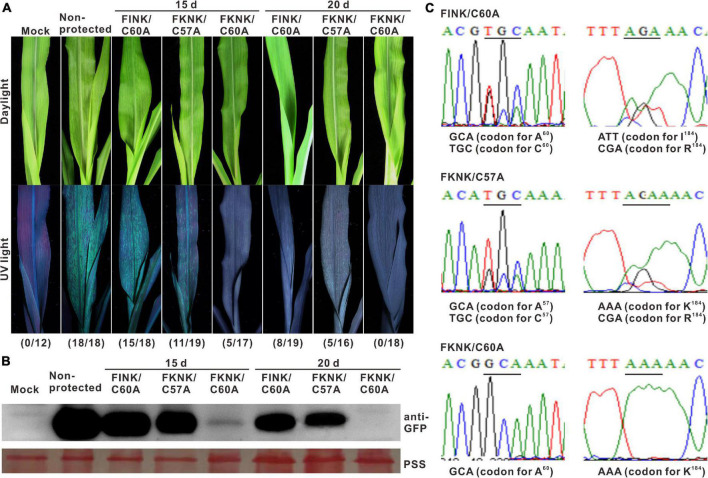
Cross protection efficacy of SCMV double-mutants against severe strain. **(A)** Symptoms of the maize plants challenged with SCMV-GFP at 20 days post-challenge inoculation with intervals of 15 or 20 dpi. Mock, the maize plants were inoculated with phosphate-buffered saline. Non-protected, the mazie plants were inoculated with pCB301-Rz vector. The numbers of symptomatic/inoculated maize plants were marked in brackets. **(B)** Western blotting analysis of the GFP accumulation levels in the maize plants challenged with wild-type SCMV-GFP at 20 days post-challenge inoculation. The ponceau S staining (PSS) shows sample loadings. **(C)** With the interval of 20 days, the sequencing result of HC-Pro RT-PCR products from the maize plants protected with FINK/C60A, FKNK/C57A or FKNK/C60A at 20 days post-challenge inoculation. The sites of point mutations in SCMV HC-Pro were underlined and the corresponding amino acid residues were indicated. The experiments were repeated thrice independently.

To test the cross-protection efficacy of three SCMV double-mutants, the first fully expanded leaves of the maize plants primarily inoculated with FINK/C60A, FKNK/C57A, and FKNK/C60A, respectively, were mechanically inoculated with wild-type SCMV-GFP with an interval of 15 or 20 days. At 20 days post challenge inoculation, the non-protected maize plants showed clear mosaic symptom under daylight and strong GFP fluorescence under UV light. With an interval of 15 days, fifteen of the eighteen maize plants protected by FINK/C60A and eleven of the nineteen maize plants protected by FKNK/C57A showed mosaic symptom and strong GFP fluorescence, whereas only five of the seventeen maize plants protected by FKNK/C60A showed mild mosaic and weak GFP fluorescence. With an interval of 20 days, eight of the nineteen maize plants protected by FINK/C60A and five of the sixteen maize plants protected by FKNK/C57A showed mild mosaic and weak GFP fluorescence, and no maize plants protected by FKNK/C60A showed mosaic symptoms and GFP fluorescence (Figure 4A). Western blotting analysis showed that GFP was accumulated in control and the treatments of FINK/C60A or FKNK/C57A, but not in the treatment of FKNK/C60A with an interval of 20 days (Figure 4B). Cross-protection tests indicated that FKNK/C60A double-mutant conferred the best cross-protection efficiency and provided complete cross-protection to the infection of wild-type SCMV with an interval of 20 days.

The HC-Pro encoding sequences of the SCMV progenies were sequenced at 20 days post-challenge inoculation. Sequencing results showed that, with the interval of 20 days, the codons of the amino acid at position 60 were GCA (codon for A^60^) and TGC (codon for C^60^), and the codons of the amino acid at position 184 were ATT (codon for I^184^) and CGA (codon for R^184^) in the treatment of FINK/C60A; the codons of the amino acid at position 57 were GCA (codon for A^57^) and TGC (codon for C^57^), and the codons of the amino acid at position 184 were AAA (codon for K^184^) and CGA (codon for R^184^) in the treatment of FKNK/C57A, indicating that mix infection of the wild type SCMV and mutant FINK/C60A or FKNK/C57A occurred in those maize plants. However, the codons of the amino acid at position 60 was GCA (codon for A^60^) and at position 184 was AAA (codon for K^184^) in the treatments of FKNK/C60A (Figure 4C), indicating the wild-type SCMV-GFP was completely excluded by FKNK/C60A mutant.

### The Stability of FKNK/C60A Mutant in Maize Plants

The HC-Pro encoding sequence from the SCMV progeny in maize plants infected with FKNK/C60A was sequenced and aligned during four serial passages by mechanical inoculation in maize plants with an interval of 15 days. After successive transfer of 4 generations, neither of the maize plants infected with FKNK/C60A double-mutant showed severe mosaic symptoms. The sequencing results indicated no mutation in the HC-Pro coding sequence from the SCMV progenies in maize plants infected with FKNK/C60A mutant after four successive passages. Thus, FKNK/C60A double-mutant were stable genetically through successive passages in maize plants.

Then the HC-Pro coding sequences from the SCMV progeny in maize plants infected with SCMV mutants at 30 dpi and 60 dpi were sequenced and aligned. At 30 dpi, one of the twenty-three maize plants infected with SCMV-HC_*FINK*_ showed severe mosaic symptoms. Sequence alignment showed that G^440^ codon (GGA) in HC-Pro was changed to R codon (AGA) (Table 1). One of the nineteen maize plants infected with SCMV-HC_*FKNK*_ showed severe mosaic symptoms. Sequence alignment showed that AAA, the codon for K at position 184 (K^184^) of HC-Pro_*FKNK*_, mutated to AGA (codon for R) (Table 1). At 60 dpi, one of the maize plants infected with SCMV-HC_*C*60*A*_-GFP or SCMV-HC_*C*57*A*_-GFP, respectively, showed severe mosaic symptoms. And GCA, the codons for A at position 57 or 60 (A^57^ or A^60^), were mutated to TGC, which is the codon for C (Table 1). One of the maize plants infected with SCMV-HC_*FINK*_ showed mild mosaic symptoms, but GAG, the codons for glutamic acid (E) at position 308 (E^308^) of HC-Pro_*FINK*_, mutated to GCG, which is the codons for A (Table 1). One of the maize plants infected with SCMV-HC_*FKNK*_ showed mild mosaic symptoms, but ATA, the codon for I at position 309 (I^309^) of HC-Pro_*FKNK*_, mutated to ATG, which is the codons for methionine (M) (Table 1). Although the spontaneous mutations of HC-Pro coding sequences from the SCMV progeny also occurred in maize plants infected with FINK/C60A, FKNK/C57A and FKNK/C60A double-mutants, all the maize plants still showed mild mosaic symptoms at 60 dpi (Table 1), indicating the attenuated symptoms caused by SCMV double-mutants were stable in maize plants at 60 dpi.

**TABLE 1:** Variability of HC-Pro genes and progeny phenotypes of SCMV mutants.

Ancestral viruses	Progeny viruses			
	30 dpi		60 dpi	
SCMV-HC_*C*57*A*_-GFP	None **0/16**	Mild mosaic	A^57^ (GCA) to C (TGC) **1/16**	Severe mosaic
SCMV-HC_*C*60*A*_-GFP	None **0/19**	Mild mosaic	A^60^ (GCA) to C (TGC) **1/19**	Severe mosaic
SCMV-HC_*FINK*_	G^440^ (GGA) to R (AGA) **1/23**	Severe mosaic	G^440^(GGA) to R (AGA) **2/23** E^308^(GAG) to A (GCG) **1/23**	Severe mosaic Mild mosaic
SCMV-HC_*FKNK*_	K^184^ (AAA) to R (AGA) **1/19**	Severe mosaic	K^184^ (AAA) to R (AGA) **3/19** I^309^ (ATA) to M (ATG) **1/19**	Severe mosaic Mild mosaic
FINK/C60A	None **0/16**	Mild mosaic	G^440^(GGA) to R (AGA) **1/16**	Mild mosaic
FKNK/C57A	None **0/15**	Mild mosaic	K^184^ (AAA) to R (AGA) **1/15**	Mild mosaic
FKNK/C60A	None **0/14**	Mild mosaic	K^184^ (AAA) to R (AGA) **1/19**	Mild mosaic

*The numbers of maize plants with spontaneous mutations/inoculated with SCMV were listed in bold.*

## Discussion

In this study, our results showed that the mutation of C^57^ and C^60^ in the zinc finger-like motif of HC-Pro affected its RSS activity and SCMV virulence. The attenuated SCMV mutants with double-mutations in the zinc finger-like and FRNK motifs were obtained. The FKNK/C60A double-mutant only caused mild symptoms in maize plants until 95 dpi and could provide complete cross-protection to the infection of wild-type SCMV with an interval of 20 days, thus it was a promising mild strain for cross-protection.

In the molecular arms race, potyvirus has evolved an effective RNA silencing suppressor (HC-Pro) to counteract the RNA silencing mechanism in plants (Anandalakshmi et al., 1998; Ivanov et al., 2016; Valli et al., 2018). Helper component-proteinase is a major virulence determinant of potyviruses and an important candidate for screening attenuated mutants (Seo et al., 2011; Huang et al., 2019; Bao et al., 2020; Cheng et al., 2020). Single or multiple amino acid mutations in the conserved motifs of HC-Pro affected potyviral virulence (Desbiez et al., 2010; Kung et al., 2014; Tuo et al., 2020). The N-terminal domain of multifunctional potyviral HC-Pro contains a zinc finger-like motif and a KITC motif (Govier and Kassanis, 1974; Atreya et al., 1992; Atreya and Pirone, 1993; Valli et al., 2014, 2018). The zinc finger-like motif in HC-Pro plays a crucial role in its self-interaction and viral virulence. The single amino acid changes C^25^ and C^53^ to glutamic (G) within the zinc finger-like motif is critical for the self-interaction of PVY HC-Pro (Urcuqui-Inchima et al., 1999). The substitution of threonine (T) at position 27 with I in HC-Pro zinc finger-like motif reduced the virulence of clover yellow vein virus (Yambao et al., 2008). The mutation of C at position 310 in the zinc finger-like motif of TVMV HC-Pro corresponding to C^57^ of SCMV HC-Pro to serine (S) has profound effects on the TVMV virulence, while the importance of C at position 313 of TVMV corresponding to C^60^ of SCMV HC-Pro remains unknown (Atreya and Pirone, 1993). This study found that the mutations on C^57^ and C^60^ in the zinc finger-like motif of HC-Pro reduced its RSS activity and SCMV virulence (Figure 2). Consistent with previous studies, our results also showed that viral symptom is correlated with the RSS activity of HC-Pro (Gal-On, 2000; Shiboleth et al., 2007; Chen L. et al., 2017). Interestingly, we noticed that the accumulation levels of HC_*C*57*A*_ and HC_*C*60*A*_ mutants were also reduced significantly (*P* < 0.05) compared to wild type HC-Pro in 16C leaf patches (Figure 2F), indicating HC-Pro mutants were more unstable than wild type HC-Pro in plants, which might be due to plant autophagy (Nakahara et al., 2012). Therefore, the attenuated virulence and RSS activity might be related to the decrease of HC-Pro accumulation. The KITC motif in HC-Pro plays a key role in aphid transmission (Atreya and Pirone, 1993; Blanc et al., 1998). Tobacco etch virus HC-Pro with the substitution of K for E in the KITC motif failed to interact with a ribosomal protein S2 in aphid heads and lost the aphid transmission activity (Fernandez-Calvino et al., 2010). The alanine (A) substitution at C^16^, C^47^ and C^49^ residues in the zinc finger-like motif of wheat streak mosaic virus HC-Pro abolished vector transmission, indicating that the conserved C residues in HC-Pro zinc finger-like motif might also be involved in aphid transmission (Young et al., 2007).

Cross-protection is an efficient method to control plant viruses. However, it has been applied only for a few crops in the field, such as *Citrus sinensis*, *Manihot esculenta*, *Theobroma cacao*, *Solanum lycopersicum*, *Cucurbita pepo*, *Cucurbita melo*, *Cucumis sativus*, and *Glycine max* (Kosaka and Fukunishi, 1993; Montasser et al., 1998; Owor et al., 2004; Zanutto et al., 2013; Ameyaw et al., 2016; Aranda and Freitas-Astúa, 2017; Agüero et al., 2018). The stability of mild strain is an important factor limiting the successful application of cross-protection (Ziebell and MacDiarmid, 2017). The spontaneous mutations increase the risk that attenuated mutants may become virulent strains (Loebenstein and Carr, 2006; Ambrós et al., 2018). Single mutation can alter the mild strain of pepino mosaic virus from mild pathotype to necrotic one in tomato and *D. inoxia* (Hasiów-Jaroszewska et al., 2011). Potyviral polymerases lack the mechanism of proofreading and repair resulting in rapid genetic changes (Roossinck, 1997). Potyvirus HC-Pro is also under continuous mutation (Torres-Barceló et al., 2008, 2009; Ambrós et al., 2018). A spontaneous mutation in HC-Pro of attenuated SCMV mutant could restore HC-Pro RSS activity and SCMV virulence (Xu et al., 2020). The attenuated mutants with two or more mutations might reduce such risk (Cong et al., 2019). The ZYMV GAC triple-mutant was stable after nine months in *Chenopodium quinoa* plants (Lin et al., 2007). The papaya plants infected with the double mutant of papaya leaf distortion mosaic virus showed symptomless leaves at 60 dpi (Tuo et al., 2020). The maize plants infected with FKNK/C60A double-mutant showed mild symptoms even at 95 dpi, and FKNK/C60A mutant should be a stable mild strain for cross-protection against severe strain. Mutations in RNA viruses occur continuously and randomly, and there are mutational hot-spots (Sanjuán and Domingo-Calap, 2016; Domingo, 2020). The 3‘ non-translated region containing numerous mutations from all cucumber mosaic virus populations is a mutational hot-spot (Schneider and Roossinck, 2001). In this study, we found that the high frequency of spontaneous mutations was observed in the central and C-terminal regions of HC-Pro (101–460 residues) from all the populations of SCMV mutants in maize plants (Table 1). Reverse mutations were not observed in HC-Pro zinc finger-like motif from the progeny of SCMV double-mutants in maize plants. Furthermore, their SCMV RNA accumulated up to about 50% of SCMV-HC_*C*57*A*_-GFP and SCMV-HC_*C*60*A*_-GFP mutants for SCMV double-mutants in the maize plants (Figures 2C, 3B). These results indicated that lower copy numbers of the double-mutants might impair the error-prone replication in HC-Pro zinc finger-like motif.

In summary, our results reveal C^57^ and C^60^ in the zinc finger-like motif of HC-Pro are involved in its RSS activity and virulence of SCMV in plants. The study also reports a promising attenuated SCMV double-mutant for cross-protection. These results provide a theoretical guide for the management of SCMV by cross-protection.

## Data Availability Statement

The original contributions presented in the study are included in the article/Supplementary Material, further inquiries can be directed to the corresponding authors.

## Author Contributions

X-JX, Y-PT, and X-DL designed the experiments and wrote the manuscript. X-JX, S-YJ, QZ, and Z-YY performed the experiments. X-JX, CG, Y-PT, and X-DL analyzed the data. All authors contributed to the article and approved the submitted version.

## Conflict of Interest

The authors declare that the research was conducted in the absence of any commercial or financial relationships that could be construed as a potential conflict of interest.

## Publisher’s Note

All claims expressed in this article are solely those of the authors and do not necessarily represent those of their affiliated organizations, or those of the publisher, the editors and the reviewers. Any product that may be evaluated in this article, or claim that may be made by its manufacturer, is not guaranteed or endorsed by the publisher.
