# Corrigendum: Development and Evaluation of Stable Sugarcane Mosaic Virus Mild Mutants for Cross-Protection Against Infection by Severe Strain

**DOI:** 10.3389/fpls.2022.956567

**Published:** 2022-06-28

**Authors:** Xiao-Jie Xu, Qing Zhu, Shao-Yan Jiang, Zhi-Yong Yan, Chao Geng, Yan-Ping Tian, Xiang-Dong Li

**Affiliations:** Shandong Province Key Laboratory for Agricultural Microbiology, Laboratory of Plant Virology, Department of Plant Pathology, College of Plant Protection, Shandong Agricultural University, Tai'an, China

**Keywords:** cross-protection, helper component-proteinase, RNA silencing suppression, spontaneous mutation, virulence, sugarcane mosaic virus

In the original article, there was a mistake in Figure 2 and Figure 3 as published. The pictures of SCMV-GFP in the second column of Figure 2B and SCMV in the second column of Figure 3A were wrongly used. We have replaced them with the correct ones. The corrected Figure 2 and Figure 3 appear below.

**Figure 2:**
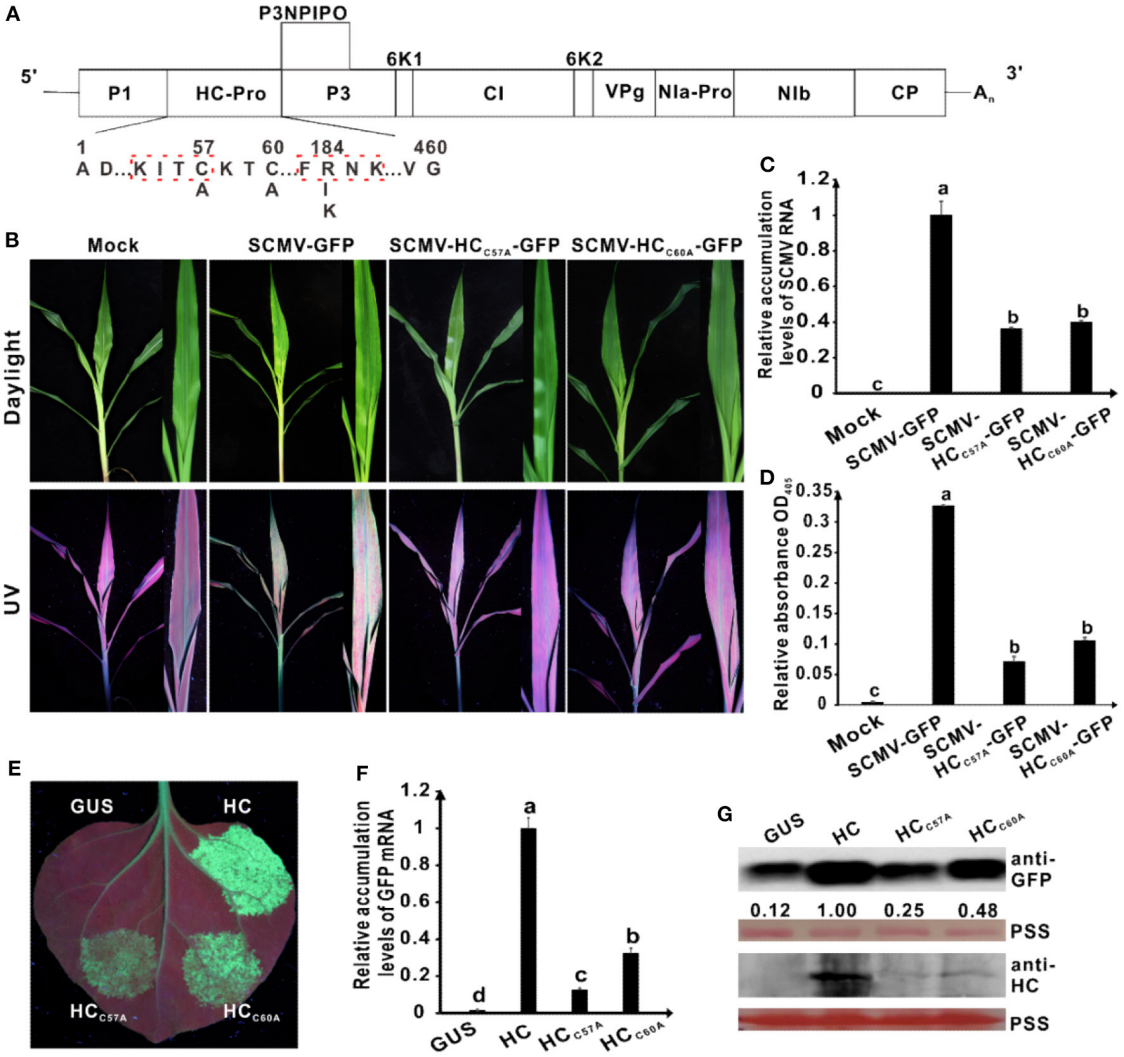
Effects of the mutations in the conserved C^57^ and C^60^ of wild type HC-Pro on its RNA silencing suppression activity and virulence of SCMV. **(A)** Genetic map of SCMV, showing the all mutations in HC-Pro. The numbers above the sequence indicate their position in SCMV HC-Pro and the letters below the sequence showed the substituted residues at that position. The highly conserved KITC and FRNK motifs in SCMV HC-Pro were marked by the red dotted boxs. **(B)** Symptoms of SCMV and two SCMV mutants in maize plants at 10 days post inoculation (dpi). The conserved C^57^ and C^60^ in wild type SCMV HC-Pro were mutated to A residues in HC-Pros of SCMV-HC_C57*A*_-GFP and SCMV-HC_C60*A*_-GFP, respectively. Mock, the maize plants inoculated with the empty vector pCB301-Rz. SCMV-GFP, the maize plants infected with wild type SCMV with *gfp* reporter gene. **(C)** The wild type and mutant SCMV RNA accumulation levels in the upper leaves of maize plants at 10 dpi. **(D)** ELISA analysis of the wild type and mutant SCMV accumulation levels in the upper leaves of maize plants at 10 dpi. **(E)** The wild type and mutants HC-Pro RSS activity in *Agrobacterium* co-infiltration assay. The *N. benthamiana* 16C leaves were infiltrated with a mixture of *Agrobacterium* cultures carrying pBin-GFP and either wild type or mutant HC-Pro and photographed under long-wavelength UV light at 3 days post agroinfiltration (dpai). The conserved C residues in wild type HC-Pro (HC) were mutated to A residues in HC_C57*A*_ and HC_C60*A*_, respectively. The GUS was used as a negative control. **(F)** The GFP mRNA accumulation levels in agroinfiltrated 16C leaf patches. **(G)** Western blotting analysis of the accumulation levels of GFP and HC in agroinfiltrated leaf patches of 16C. The ponceau S staining (PSS) shows sample loadings. Band intensities were measured using the ImageJ software. Numbers indicate the accumulation levels of SCMV CP normalized to PSS staining. Error bars indicate the means ± standard deviation of three replicates. Statistical significance was determined by employing *Tukey* multiple range test for between-group comparisons. Different letters indicate significant differences (*P* < 0.05). The same below. The experiments were repeated thrice independently.

**Figure 3:**
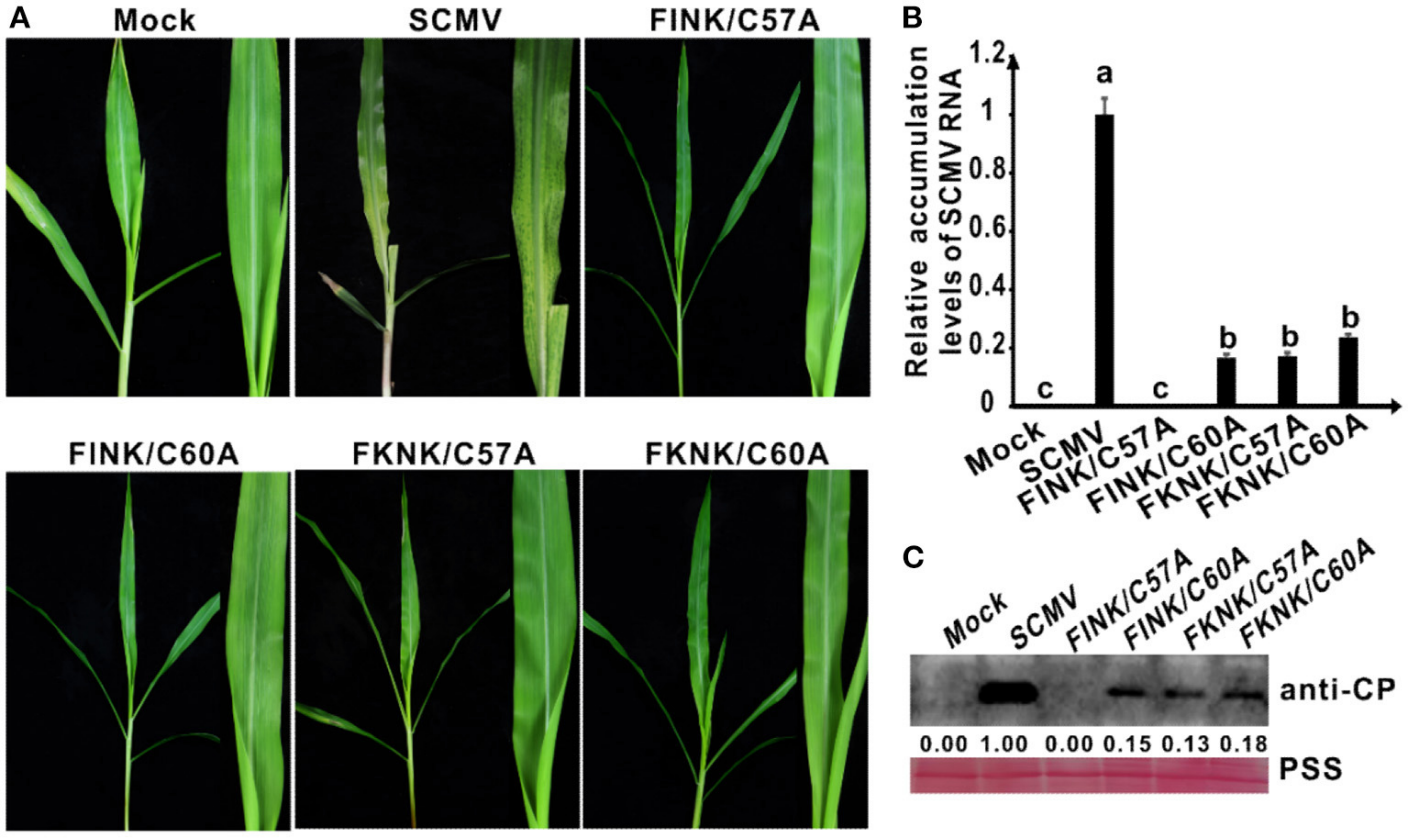
Symptoms and accumulation levels of SCMV double-mutants in maize plants. **(A)** Symptoms of wild-type SCMV and its double-mutants in maize plants at 10 dpi. Mock, the maize plants inoculated with the empty vector pCB301-Rz. SCMV, the maize plants infected with wild-type SCMV. FINK/C60A, the conserved C^60^ was mutated to A residues and R^184^ was mutated to I residues in SCMV HC-Pro. FKNK/C57A, the conserved C^57^ were mutated to A residues and R^184^ were mutated to K residues in SCMV HC-Pro. FKNK/C60A, the conserved C^60^ was mutated to A residues and R^184^ were mutated to K residues in SCMV HC-Pro. **(B)** The wild-type and mutant SCMV RNA accumulation levels in the upper leaves of maize plants at 10 dpi. **(C)** Western blotting analysis of the wild type and mutant SCMV accumulation levels in the upper leaves of maize plants at 10 dpi. The ponceau S staining (PSS) shows sample loadings. Band intensities were measured using the ImageJ software. Numbers indicated the accumulation levels of SCMV CP normalized to PSS staining. The experiments were repeated thrice independently. The statistical analyses as above.

The authors apologize for this error and state that this does not change the scientific conclusions of the article in any way. The original article has been updated.

## Publisher's Note

All claims expressed in this article are solely those of the authors and do not necessarily represent those of their affiliated organizations, or those of the publisher, the editors and the reviewers. Any product that may be evaluated in this article, or claim that may be made by its manufacturer, is not guaranteed or endorsed by the publisher.

