# Supplementary material for: Development and Evaluation of Stable Sugarcane Mosaic Virus Mild Mutants for Cross-Protection Against Infection by Severe Strain
Source: Front Plant Sci. 2021 Dec 17;12:788963. doi: 10.3389/fpls.2021.788963 (PMC8718998; doi:10.3389/fpls.2021.788963)
Supplement: Supplementary file 1 [file Table_2.DOCX]

**Supplemental Table 1: The name and sequence of the primers used in this study**

| Purpose | Primer name | Primer sequence (5'-3') |
| --- | --- | --- |
| SCMV mutants | SCMV-HC_R184I_-F | GGATTCATTTATTAACAAGATCTCACCAAAGAGCACGATC |
|  | SCMV-HC_R184I_-R | GATCTTGTTAATAAATGAATCCAATGTGTCCGTTTTAAGTGATTC |
|  | SCMV-HC_R184K_-F | ATTCATTTAAAAACAAGATCTCACCAAAGAGCACGATCAATG |
|  | SCMV-HC_R184K_-R | GAGATCTTGTTTTTAAATGAATCCAATGTGTCCGTTTTAAGTG |
|  | SCMV-HC_C57A_-F | CAAAATTACAGCAAAAACGTGCAATATTGATGATCTGGAACTGTCAGATG |
|  | SCMV-HC_C57A_-R | CACGTTTTTGCTGTAATTTTGAAAGTTGAATGGAATAGGATTTCTAACAGTG |
|  | SCMV-HC_C60A_-F | GCAAAACGGCAAATATTGATGATCTGGAACTGTCAGATGATGAATTTG |
|  | SCMV-HC_C60A_-R | TCAATATTTGCCGTTTTGCATGTAATTTTGAAAGTTGAATGGAATAGGAT |
| Real-time RT-PCR | SCMV-CP-qRT-F | GGCGAGACTCAGGAGAATACA |
|  | SCMV-CP-qRT-R | ACACGCTACACCAGAAGACACT |
|  | *ZmUbi*-qRT-F | GGAAAAACCATAACCCTGGA |
|  | *ZmUbi*-qRT-R | ATATGGAGAGAGGGCACCAG |
|  | *actin*-qRT-F | CTGATGAAGATACTCACAGAAAGAG |
|  | *actin*-qRT-R | CAGGATACGGGGAGCTAATG |
|  | GFP-qRT-F | GTGGAGAGGGTGAAGGTGAT |
|  | GFP-qRT-R | CGGATAACGGGAAAAGCATTGA |
| Transient expression vectors | pBin-SCMV-HC-F | CGGGATCCATGGCTGATCCACAAGCGAATAG |
|  | pBin-SCMV-HC-R | GGAGCTCTTATCCCACTATATATTCACGCATCTC |

The underlined letters represented the mutation sites or multiple cloning sites.
